# Utilization Pattern for Eculizumab Among Children With Hemolytic Uremic Syndrome

**DOI:** 10.3389/fped.2021.733042

**Published:** 2021-10-05

**Authors:** Saritha Ranabothu, Clare C. Brown, Richard Blaszak, Rachel Millner, Kristen Rice Moore, Parthak Prodhan

**Affiliations:** ^1^Department of Pediatrics, Pediatric Nephrology, Arkansas Children's Hospital, University of Arkansas for Medical Sciences, Little Rock, AR, United States; ^2^Health Policy and Management, Fay W. Boozman College of Public Health, University of Arkansas for Medical Sciences, Little Rock, AR, United States; ^3^College of Medicine, University of Arkansas for Medical Sciences, Little Rock, AR, United States; ^4^Pediatrics, Pediatric Cardiology/Pediatric Critical Care, Arkansas Children's Hospital, University of Arkansas for Medical Sciences, Little Rock, AR, United States

**Keywords:** hemolytic uremic syndrome, pediatrics—children, pediatric health information system (PHIS), multiorgan involvement, eculizumab

## Abstract

**Background:** Hemolytic uremic syndrome (HUS) is a complex disease with multi-organ involvement. Eculizumab therapy is recommended for treatment of complement mediated hemolytic uremic syndrome (cHUS). However, there are few studies evaluating eculizumab therapy among children with HUS. The primary objectives of the study were to describe and identify factors associated with eculizumab therapy in children with HUS.

**Design/Methods:** This large, retrospective, multi-center, cohort study used the Pediatric Health Information System (PHIS) database to identify the index HUS-related hospitalization among patients ≤18 years of age from September 23, 2011 (Food and Drug Administration approval date of eculizumab) through December 31, 2018. Multivariate analysis was used to identify independent factors associated with eculizumab therapy during or after the index hospitalization.

**Results:** Among 1,885 children included in the study, eculizumab therapy was noted in 167 children with a median age of 3.99 years (SD ± 4.7 years). Eculizumab therapy was administered early (within the first 7 days of hospitalization) among 65% of children who received the drug. Mortality during the index hospitalization among children with eculizumab therapy was 4.2 vs. 3.0% without eculizumab therapy (*p* = 0.309). Clinical factors independently associated with eculizumab therapy were encephalopathy [odds ratio (OR) = 3.09; *p* ≤ 0.001], seizure disorder (OR = 2.37; *p* = 0.006), and cardiac involvement (OR = 6.36, *p* < 0.001).

**Conclusion(s):** Only 8.9% of children received eculizumab therapy. Children who presented with neurological and cardiac involvement with severe disease were more likely to receive eculizumab therapy, and children who received therapy received it early during their index hospitalization. Further prospective studies are suggested to confirm these findings.

## Introduction

Hemolytic uremic syndrome (HUS) is a thrombotic microangiopathy defined by the classic triad of non-immune microangiopathic hemolytic anemia, thrombocytopenia, and acute renal failure. Hemolytic uremic syndrome can be broadly classified into hereditary and secondary HUS based on etiology. The most common cause of HUS is secondary to shiga toxin-producing *Escherichia Coli* (STEC) accounting for 90% of cases in children. Hereditary causes of HUS, formerly known as atypical HUS, typically involve mutations in the alternative pathway of complement, consequently they are often referred to as complement mediated hemolytic uremic syndrome (cHUS). Complement mediated hemolytic uremic syndrome may be responsible for 50–60% of non-STEC HUS (1). Unfortunately, the distinction between STEC HUS and non-STEC HUS is not always clear, as about 5% of children with STEC HUS may not present with diarrhea, and 30% of children with non-STEC HUS will present with diarrhea (2). Additionally, 40–50% of children with cHUS, may have normal genetic testing (3).

Following its US Food and Drug Administration (FDA) approval in 2011, eculizumab, a long-acting humanized monoclonal antibody targeted against complement C5, remains the recommended treatment for cHUS (2, 4). However, despite its therapeutic promise (5, 6), therapy decisions are hampered by the lack of timely diagnostic testing and a delay in diagnosis, especially in children with incomplete or uncertain initial clinical presentation. In this setting, despite accumulating evidence for the efficacy of eculizumab therapy (5, 7–11), it is unclear which clinical characteristics in children with HUS may drive the clinical decision to administer eculizumab therapy.

We add to this literature by analyzing the Pediatric Health Information System (PHIS) database, a large multicenter pediatric database, to investigate eculizumab therapy in children with HUS. As such, the primary objectives of the study were to describe and identify clinical characteristics that are associated with eculizumab therapy among children with HUS. Knowledge of these clinical findings may help identify children with HUS who would most benefit from eculizumab therapy and reduce delays in treatment due to diagnostic uncertainty.

## Methods

This study was deemed to be non-human subject research by the University of Arkansas for Medical Sciences Institutional Board Review. All methods were carried out in accordance with relevant guidelines and regulations. No experimental protocols were used.

### Data Source

The data for this retrospective cohort study of children with HUS came from PHIS, an administrative database that contains inpatient data from 44 US not-for-profit, tertiary care children's hospitals in North America (12). Institutions are affiliated with the Child Health Corporation of America (CHCA; Shawnee Mission, KS) and account for 20% of all tertiary care children's hospitals in the US. Participating hospitals provide discharge data, diagnoses coded with the International Classification of Diseases, Ninth Revision (ICD-9) or Tenth Revision (ICD-10), and procedural information using Correct Procedures Terminology (CPT) codes. Data are de-identified at the time of submission and are subjected to a number of reliability and validity checks before being added into the database. The diagnoses, procedures, and corresponding codes used in this study are provided in Supplemental Material eTable 1.

### Study Population

**Figure 1:**
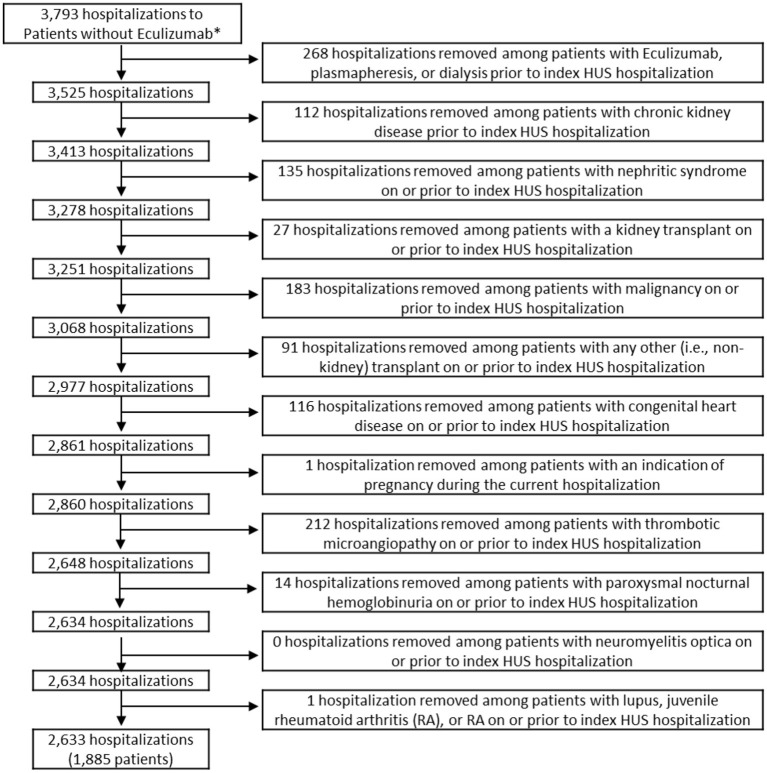
Inclusion and exclusion criteria of patients who did not receive eculizumab. *Hospitalizations were limited to those among patients aged ≤18 who were discharged after 9/23/2011. Patients in the non-eculizumab group had no eculizumab during or after the index HUS visit. HUS, hemolytic uremic syndrome.

**Figure 2:**
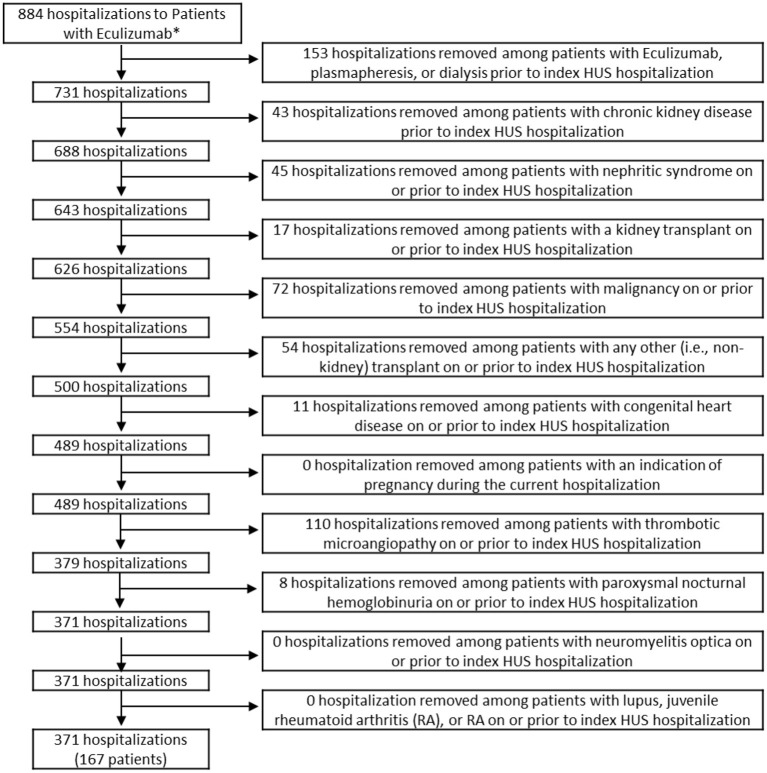
Inclusion and exclusion criteria of patients who received eculizumab. *Hospitalizations were limited to those among patients aged ≤18 who were discharged after 9/23/2011. Patients in the eculizumab group had eculizumab during or after the index HUS visit. HUS, hemolytic uremic syndrome.

The study sample included children 18 years old or younger with a diagnosis code of HUS who were discharged from a PHIS participating hospital between September 23, 2011 (date of FDA approval for eculizumab) and December 31, 2018. The final sample included 1,885 children. All exclusions are indicated in the flow diagram in Figures 1, 2. Data for demographics, clinical characteristics, and various diagnoses and procedures were abstracted. The primary outcome measure for the study was whether the child received eculizumab therapy during or after their index HUS hospitalization.

### Statistical Analysis

Descriptive statistics included *t*-tests for continuous variables and Wald chi-square tests for categorical variables to test for differences between characteristics of children with and without eculizumab therapy. Fisher's exact tests were used for two analyses with small cell sizes, which are identified in the Results section. Multivariable logistic regression analysis was used to assess factors associated with initiation of eculizumab therapy during or after the index HUS hospitalization. Covariates selected for inclusion in the multivariable models included demographic characteristics, comorbidities, and procedures. Given the noted unadjusted associations between eculizumab therapy with gastrointestinal conditions, neurological conditions, and cardiac conditions, we conducted three separate logistic regression models to comprehensively assess the impact of these comorbid conditions. Specifically, we assessed (1) the associations of individual conditions from the three condition categories (i.e., gastrointestinal, neurological, and cardiac), (2) associations of binary variables indicating whether a child had specific conditions (e.g., seizure disorder) within one of the three condition categories, and (3) an additive effect model constructed by adding the number of condition categories present for a child, ranging from 0 to 3, with 0 representing that the child had no gastrointestinal, neurological, or cardiac conditions, and 3 representing that the child had a condition from all three categories (*post-hoc* decision). All statistical analyses were conducted using STATA Version 16 (StataCorp LLC, College Station, TX), and graphs were created using Tableau 2019.4 (Tableau Software LLC, Seattle, WA). All tests were two-sided assuming a *p*-value < 0.05 as statistically significant.

## Results

**Figure 3:**
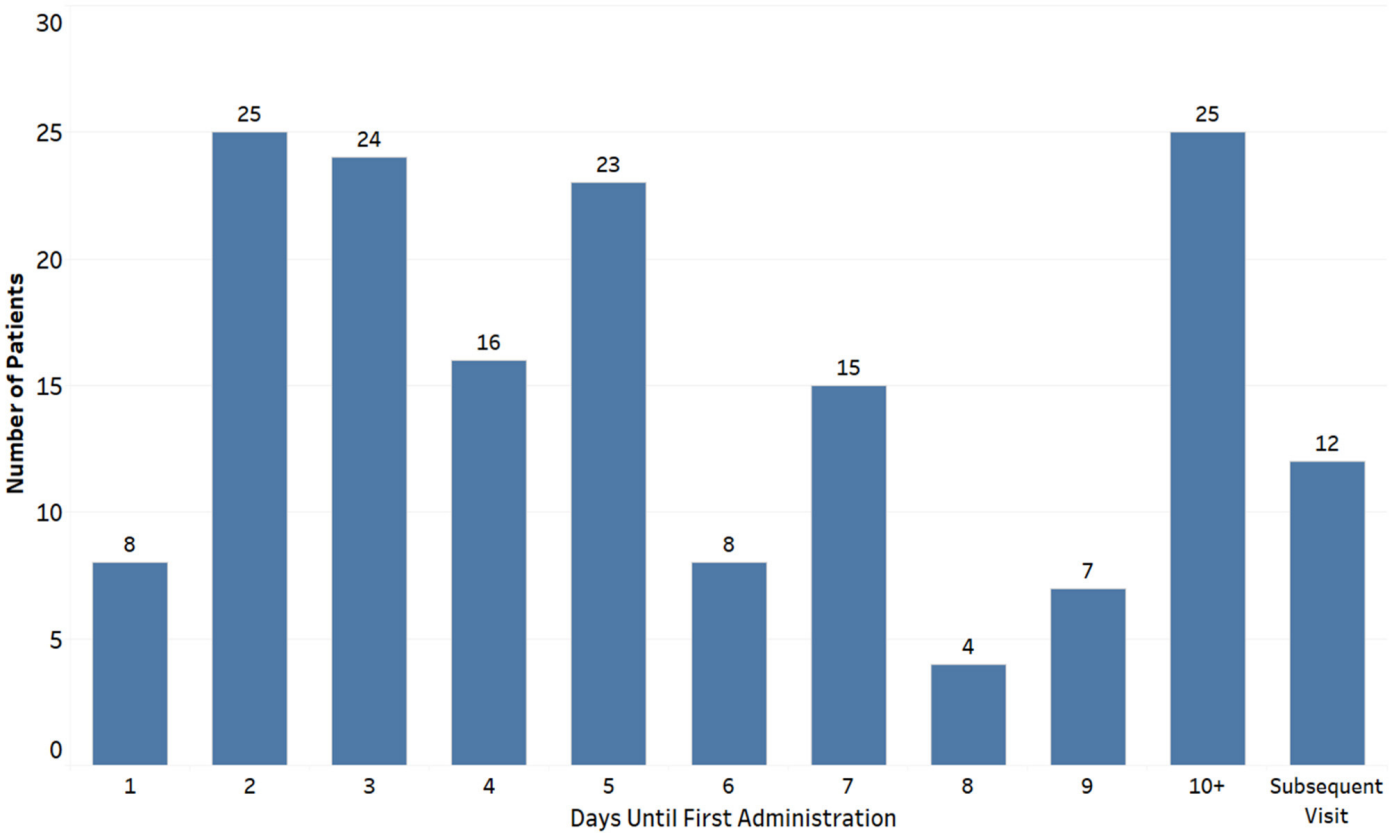
Day of eculizumab administration after index hospitalization among children with hemolytic uremic syndrome receiving eculizumab therapy (*n* = 167).

Among the 1,885 children with HUS included in this study, 167 children (8.9%) received eculizumab therapy on or after their index hospitalization (Eculizumab group) and had a total of 371 hospitalizations. There were 1,718 children who did not receive eculizumab therapy (Non-eculizumab group), with a total of 2,262 hospitalizations. Among children who received eculizumab therapy, 65% of the children initiated therapy within the first 7 days of the admission of the index hospitalization (Figure 3).

At the index hospitalization, the median age for the Eculizumab group was 3.99 years with 52.1% of children under 5 years of age (Table 1). During index hospitalization, children in the Eculizumab group were more likely to have gastrointestinal (*p* < 0.001), neurological (*p* < 0.001), or cardiac (*p* < 0.001) conditions, shock (*p* < 0.001) or sepsis (*p* < 0.001), and/or procedures such as gastrointestinal surgery (*p* = 0.03), gastrostomy (*p* < 0.001), pleural drainage (*p* < 0.001), or mechanical ventilation (*p* < 0.001) compared to the Non-eculizumab group. During index admission, dialysis (*p* = 0.008) or plasmapheresis (*p* < 0.001) were more frequently used in the Eculizumab group. Additionally, more children “acquired” a diagnosis of chronic kidney disease in the Eculizumab group (*p* < 0.001). However, children in the Non-eculizumab group were more likely to have enteritis (58.7%) or Shiga toxin (STEC; 40.8%) compared to the Eculizumab group (*p* < 0.001).

**Table 1:** Descriptive statistics at index hospitalization for children with hemolytic uremic syndrome with and without eculizumab therapy on or after the index hospitalization.

	**Non-eculizumab group**		**Eculizumab group**		***p*-value**
	**(*****N*** **= 1,718)**		**(*****N*** **= 167)**		
	***n***	**%**	***n***	**%**	
Age					
<1 year	158	9.2	26	15.6	<0.001
1– <5 years	859	50.0	61	36.5	
≥5 years	707	40.8	80	47.9	
Gender					
Female	908	52.9	99	59.3	0.112
Male	810	47.2	68	40.7	
Race					
White	1,307	76.1	114	68.3	0.139
Black	101	5.9	13	7.8	
Asian	50	2.9	8	4.8	
Other	260	15.1	32	19.2	
Clinical comorbidities					
Any gastrointestinal condition^a^	466	27.1	72	43.1	<0.001
Any neurologic condition	166	9.7	61	36.5	<0.001
Specific neurologic conditions					
Seizure disorder	58	3.4	27	16.2	<0.001
Encephalopathy	99	5.8	44	26.4	<0.001
Cerebral edema/compression	16	1.0	6	4.6	0.002
Other neurologic conditions^b^	66	3.8	20	12.0	<0.001
Any cardiac condition	645	37.5	107	64.1	<0.001
Specific cardiac conditions					
Hypertension	606	35.3	94	56.3	<0.001
Myocarditis/Cardiomyopathy/congestive heart failure	21	1.2	21	12.6	<0.001
Other cardiac conditions^c^	76	4.4	25	15.0	<0.001
Chronic kidney disease^d^	90	5.2	23	13.8	<0.001
Plasmapheresis	52	3.0	17	10.2	<0.001
Dialysis	528	30.7	68	40.7	0.008
Sepsis	167	9.7	31	18.6	<0.001
Shock	40	2.3	15	9.0	<0.001
Shiga toxin positive	700	40.8	44	26.4	<0.001
Pneumococcal infection	50	2.9	8	4.8	0.179
Enteritis	1009	58.7	58	34.7	<0.001
Additive gastrointestinal, neurologic, and cardiac effect^e^					
0 (no effects)	787	45.8	28	16.8	<0.001
1	627	36.5	62	37.1	
2	262	15.3	53	31.7	
3	42	2.4	24	14.4	
Procedures					
Gastrointestinal surgery	24	1.4	6	3.6	0.03
Gastrostomy	15	0.9	10	6.0	<0.001
Pleural drain	41	2.4	10	6.0	0.006
Endotracheal intubation	211	12.3	49	29.3	<0.001

a *Any gastrointestinal condition includes gastrointestinal ulceration, hemorrhage, volvulus, intussusception, inflammatory or ischemic bowel disease, eosinophilic colitis, ileostomy or fistulas, gastro paresis, persistent vomiting, and liver or gall bladder disease*.

b *Other neurological conditions include meningitis, intracerebral hemorrhage, cerebral infarct, hemiplegia, and anoxic brain*.

c *Other cardiac conditions include pericardial disease, endocarditis, cardiac arrest, valve disorders, arrhythmia, and heart block*.

d *Note that children with chronic kidney disease prior to the index hospitalization were excluded*.

e *A variable was constructed to evaluate the potential additive effect of having multiple conditions among gastrointestinal, neurologic, and cardiac categories. This variable was constructed by adding three binary variable indicators for “any gastrointestinal condition,” “any neurologic condition,” and “any cardiac condition”*.

During the index hospitalization, the average hospital length of stay (LOS) was significantly longer for the Eculizumab group (24.0 days) compared to the Non-eculizumab group (15.7 days, *p* < 0.001; Table 2). A higher percentage of the children required an intensive care unit (ICU) stay in the Eculizumab group (73.7%) compared to the Non-eculizumab group (47.0%; *p* < 0.001) or had at least one subsequent hospitalization (38.3% in the Eculizumab group compared to 10.8% in the Non-eculizumab group; *p* < 0.001). A larger percentage of children received dialysis (40.7% in the Eculizumab group compared to 30.7% in the Non-eculizumab group) or plasmapheresis (10.2% in the Eculizumab group compared to 3.0% in the Non-eculizumab group).

**Table 2:** Outcomes of index hospitalizations among children with hemolytic uremic syndrome, by administration and timing of eculizumab therapy.

	**Overall index hospitalizations**			**Index hospitalizations by quartile^a^**				
	**(*****N*** **= 1,885)**			**(*****N*** **= 155)**				
	**No Eculizumab**	**Eculizumab**	***p*-value**	**Q1**	**Q2**	**Q3**	**Q4**	***p*-value**
	**(*n* = 1,718)**	**(*n* = 167)**		**(*n* = 33)**	**(*n* = 40)**	**(*n* = 46)**	**(*n* = 36)**	
Hospital LOS	15.7	24.0	<0.001	21.1	20.3	22.3	35.9	0.001
ICU LOS (including zeros; %)	6.3	10.1	0.018	7.8	8.0	9.8	16.5	0.008
Any ICU (%)	47.0	73.7	<0.001	66.7	77.5	73.9	88.9	0.166
Mortality (%)	3.0	4.2	0.409	3.0	5.0	4.4	5.6	0.963
Subsequent hospitalization events (%)	10.8	38.3	<0.001	39.4	30	30.4	36.1	0.792
Received plasmapheresis (%)	3.0	10.2	<0.001	3.0	15.0	6.5	19.4	0.095
Received dialysis (%)	28.4	39.5	0.003	22.3	47.5	34.8	55.6	0.068

*ICU, intensive care unit; LOS, length of stay; Q, quartile*.

a *Timing of eculizumab therapy: Q1, therapy initiated on days 1–2 of index hospitalization; Q2, days 3–4; Q3, days 5–7; Q4, days 8 and later*.

To assess outcome based on the day of initiation of eculizumab therapy after hospitalization, the cohort was divided into quartiles among the 155 children who received therapy during their index hospitalization (Q1, day 1–2 of hospitalization; Q2, day 3–4; Q3, 5–7 days; and Q4, day 8 or later; Table 2). Children who received eculizumab later had longer hospital LOS and/or ICU LOS. No significant difference was noted in mortality, percentage of children who received dialysis, or percentage of children who received plasmapheresis.

Table 3 provides the results of the multivariate logistic regressions that assessed factors associated with initiation of eculizumab therapy (Supplemental Material eTable 2). Model 1 provides the odds of eculizumab therapy associated with specific conditions within organ system involvement. The conditions that were most strongly associated with eculizumab administration were seizure disorder [odds ratio (OR) = 2.74; 95% CI, 1.50–5.00], encephalopathy (OR = 3.14; 95% CI, 1.82–5.43), and myocarditis/cardiomyopathy/congestive heart failure (OR = 6.99; 95% CI, 3.30–14.80). Model 2 provides the odds of eculizumab therapy associated with condition categories within organ system involvement and indicates that the absence of enteritis (OR = 3.93; 95% CI,: 2.12–7.28; *p* < 0.001) and the presence of any neurological (OR = 3.81; 95% CI, 2.50–5.81; *p* < 0.001) or cardiac condition (OR = 2.63; 95% CI, 1.82–3.81; *p* < 0.001) were associated with increased probability of eculizumab therapy. Model 3 demonstrates an additive effect of having a condition from more than one of the condition categories, representing multi-organ involvement (i.e., gastrointestinal, neurological, and/or cardiac). This was indicated by an incremental increase in the OR among children with 1-, 2-, or 3- organ involvement relative to children with no organ involvement. Specifically, 2-organ involvement (OR = 5.16; 95 % CI, 3.06-8.71; *p* < 0.001) and 3-organ involvement (OR = 14.00; 95% CI, 6.83-28.71; *p* < 0.001) were strongly associated with eculizumab administration.

**Table 3:** Clinical factors associated with eculizumab administration.

	**Individual conditions (Model 1)**			**Grouped conditions (Model 2)**			**Additive effect of grouped conditions^a^ (Model 3)**		
	**OR**	**95% CI**	***p*-value**	**OR**	**95% CI**	***p*-value**	**OR**	**95% CI**	***p*-value**
Age									
<1 year	0.66	(0.37, 1.20)	0.177	0.69	(0.39, 1.23)	0.209	0.71	(0.41, 1.25)	0.240
1– <5 years	1.24	(0.69, 2.23)	0.480	1.33	(0.76, 2.35)	0.322	1.31	(0.75, 2.30)	0.345
≥5 years	Reference								
Race									
White	Reference								
Black	0.96	(0.48, 1.91)	0.907	1.03	(0.53, 2.02)	0.922	1.04	(0.54, 2.02)	0.906
Asian	1.82	(0.75, 4.42)	0.183	1.90	(0.80, 4.53)	0.147	1.89	(0.81, 4.45)	0.144
Other	1.22	(0.76, 1.96)	0.404	1.27	(0.80, 2.00)	0.307	1.30	(0.83, 2.03)	0.257
Male	0.76	(0.53, 1.08)	0.125	0.72	(0.51, 1.03)	0.072	0.73	(0.51, 1.03)	0.075
Plasmapheresis	1.72	(0.85, 3.47)	0.133	1.73	(0.89, 3.39)	0.108	1.88	(0.97, 3.64)	0.060
Dialysis	1.11	(0.75, 1.65)	0.592	1.08	(0.73, 1.59)	0.690	1.06	(0.72, 1.56)	0.767
Sepsis	0.82	(0.46, 1.47)	0.502	0.83	(0.47, 1.45)	0.508	0.80	(0.46, 1.38)	0.418
Shock	1.93	(0.89, 4.18)	0.096	2.40	(1.17, 4.91)	0.017	2.36	(1.15, 4.82)	0.019
Pleural drain	1.73	(0.71, 4.23)	0.228	1.88	(0.81, 4.33)	0.140	1.76	(0.77, 4.05)	0.180
Endotracheal tube	1.22	(0.74, 2.02)	0.441	1.30	(0.81, 2.11)	0.278	1.51	(0.94, 2.43)	0.089
Pneumococcal	0.82	(0.32, 2.10)	0.674	0.74	(0.30, 1.81)	0.508	0.81	(0.33, 1.97)	0.636
Any gastrointestinal condition^b^	1.38	(0.94, 2.01)	0.096	1.36	(0.94, 1.97)	0.099			
Gastrointestinal surgery	1.55	(0.49, 4.85)	0.453	1.66	(0.59, 4.69)	0.335	1.80	(0.65, 4.95)	0.258
Shiga toxin positive	1.16	(0.59, 2.28)	0.661	1.28	(0.67, 2.45)	0.462	1.29	(0.68, 2.47)	0.438
Not enteritis	3.96	(2.11, 7.43)	<0.001	3.93	(2.12, 7.28)	<0.001	3.94	(2.13, 7.29)	<0.001
Any neurologic condition				3.81	(2.50, 5.81)	<0.001			
Seizure	2.74	(1.50, 5.00)	0.001						
Encephalopathy	3.14	(1.82, 5.43)	<0.001						
Cerebral edema/compression	1.18	(0.38, 3.68)	0.781						
Other neurologic conditions^c^	1.11	(0.55, 2.24)	0.761						
Any cardiac condition				2.63	(1.82, 3.81)	<0.001			
Hypertension	2.05	(1.42, 2.98)	<0.001						
Myocarditis/ cardiomyopathy/ congestive heart failure	6.99	(3.30, 14.80)	<0.001						
Other cardiac conditions^d^	1.25	(0.66, 2.38)	0.489						
Number of gastrointestinal, neurologic, and cardiac conditions									
0							Reference		
1							2.92	(1.82, 4.68)	<0.001
2							5.16	(3.06, 8.71)	<0.001
3							14.00	(6.83, 28.71)	<0.001

*CI, confidence interval; OR, odd ratio*.

a *A variable was constructed to evaluate the potential additive effect of having multiple conditions among gastrointestinal, neurologic, and cardiac conditions. This variable was constructed by adding three binary variable indicators for “any gastrointestinal condition,” “any neurologic condition,” and “any cardiac condition”*.

b *Any gastrointestinal condition includes gastrointestinal ulceration, hemorrhage, volvulus, intussusception, inflammatory or ischemic bowel disease, eosinophilic colitis, ileostomy or fistulas, gastroparesis, persistent vomiting, liver, or gall bladder disease*.

c *Other neurological conditions include meningitis, intracerebral hemorrhage, cerebral infarct, hemiplegia, and anoxic brain*.

d *Other cardiac conditions include pericardial disease, endocarditis, cardiac arrest, valve disorders, arrhythmia, and heart block*.

Logistic regression was done after excluding 12 children who first received eculizumab on a subsequent hospitalization. This sensitivity analysis did not yield meaningfully different results (Table 2).

## Discussion

To our knowledge, this investigation is the largest published systematic assessment of eculizumab therapy among children with HUS. Children with severe disease, as evidenced by neurological and cardiac involvement, are more likely to receive eculizumab therapy during their hospitalization for HUS.

Our study found that clinicians were more likely to initiate eculizumab therapy among children with severe multisystem disease, as indicated by the presence of extra-renal manifestations such as neurologic and cardiac manifestations. Our results are similar to a recent global registry report on eculizumab therapy, which indicated that children with cHUS had more frequent extra-renal manifestations (7). Furthermore, extra-renal manifestations have been found in about 20% of cHUS cases, many of which can have a severe clinical presentation with sequelae (13, 14). In our study, extra-renal manifestations requiring dialysis or plasmapheresis were not clinical practices identified as likely being triggers for initiating eculizumab therapy. This could be a result of the relatively small percent of children who received dialysis or plasmapheresis overall and the likelihood that adjusting for other clinical characteristics may reduce the associated OR of the two procedures. Previous studies have reported wide variation in median time (6–547 days) from disease onset to eculizumab administration (7, 8, 15). The current study showed that 65% of children in the Eculizumab group received the drug during the first 7 days of their index hospitalization with a median time of 5 days. The median time of eculizumab administration from the day of index hospitalization among children who did not receive it on the index admission was 237 days.

Children who were administered eculizumab after 7 days stayed in the hospital and the ICU longer compared to children who were administered eculizumab earlier in their hospitalization. Due to study limitation, we could not conclude that early initiation lead to faster recovery; this may also mean that the decision to administer eculizumab was made due to prolonged recovery or worsening of disease. Though there is some evidence from previous studies indicating improved outcomes with early initiation of eculizumab therapy (i.e., better renal recovery, lower rates of dialysis, reduced hospital and ICU LOSs, lower costs, improved quality of life in cHUS, and improved neurological outcomes in STEC HUS) (10, 16–18), larger controlled trials are still necessary to fill knowledge gaps regarding early eculizumab administration.

Clinical presentations for STEC HUS, cHUS, and thombotic thrombocytopenic purpura (TTP) overlap (4). About 5% children with STEC HUS may not present with diarrhea (the hallmark indicator of STEC HUS) (19), and 30% of children with non-STEC HUS may have diarrhea on initial presentation (2). In the present study, about one-third of children (34.7%) in the Eculizumab group had enteritis, and among those with enteritis, 26.4% were positive for STEC. Current consensus suggests initiating eculizumab therapy soon after ruling out STEC HUS and TTP; however, in our study, with an STEC positivity of 39%, only approximately 8% of all HUS children received eculizumab therapy on their index HUS hospitalization. Of note is that at the index hospitalization, 41% of children who did not receive eculizumab therapy were STEC positive, compared to 26% of children who did receive eculizumab therapy (2, 4). Latency of STEC HUS after enterocolitis can often lead to negative STEC testing, so negative STEC does not prompt further testing for hereditary HUS or initiation of eculizumab therapy. Our study findings indicate that clinicians may be relying on clinical factors such as severity of extra-renal manifestations to initiate eculizumab therapy than waiting to establish underlying disease process. Given the lack of rigorous ascertainment of disease process in this administrative database, the utilization of severity of extra-renal manifestations to define clinical decision-making to start complement-targeted therapy may be less than ideal. Prospective studies are required to definitively ascertain clinical practice and to define if a clinical predictive score could be useful to guide eculizumab therapy (20). It would be most beneficial clinically to identify patient characteristics that favor early identification and that are not subject to quick laboratory turnaround time.

There are a number of limitations to our study. This study used an administrative dataset that was collected for clinical and billing purposes rather than for research. The use of the PHIS administrative database provides a large dataset, which ultimately allows for a large enough sample size for analyses on such relatively rare events, but the PHIS is limited by the availability of some types of data, such as laboratory values or mortality outside of the inpatient setting. Lack of laboratory data as well as specificity with current diagnostic codes limits accurate classification of STEC HUS vs. cHUS: however, we adjusted for three types of infections in our adjusted models. The coding in the database indicates a smaller percentage of children with STEC positive HUS than what has been suggested in previously published reports. As such, we were unable to further investigate among HUS subtypes. Additionally, the PHIS data does not contain information on all types of safety and adverse events and investigations such as electroencephalogram and imaging findings. Despite these limitations, this study provides the largest evaluation of eculizumab therapy in children with HUS by using a national dataset from the majority of metropolitan areas in the US. This study provides information that can serve as a foundation for further investigation through a prospective study design or by data linkage with other national databases and for individual-level clinical decision-making.

Children with HUS and extra-renal manifestations are more likely to receive eculizumab, and children who received eculizumab therapy received it soon after the diagnosis of HUS. This emphasizes the need to conduct a prospective study to evaluate the benefit of eculizumab therapy in children with extra-renal manifestation especially when administered early in the disease courses of both cHUS and STEC HUS.

## Data Availability Statement

The data analyzed in this study is subject to the following licenses/restrictions: under a data user agreement we can't share any data from the dataset other than that which is presented in this paper and Supplementary Material. Any questions regarding access to the dataset should be directed to senior author Parthak Prodhan (ProdhanParthaK@uams.edu).

## Ethics Statement

The studies involving human participants were reviewed and approved by the University of Arkansas for Medical Sciences Institutional Board Review. Written informed consent from the participants' legal guardian/next of kin was not required to participate in this study in accordance with the national legislation and the institutional requirements.

## Author Contributions

SR, PP, and RB contributed to conception and design of the study. CB organized the database and performed the statistical analysis. SR wrote the first draft of the manuscript. PP, RB, CB, RM, and KR wrote sections of the manuscript. All authors contributed to manuscript revision, read, and approved the submitted version.

## Funding

The project described was supported by the Translational Research Institute through the National Center for Advancing Translational Sciences of the National Institutes of Health (NIH; Award ID: UL1 TR003107). The content is solely the responsibility of the authors and does not necessarily represent the official views of the NIH. The funding agency did not participate in the work.

## Conflict of Interest

The authors declare that the research was conducted in the absence of any commercial or financial relationships that could be construed as a potential conflict of interest.

## Publisher's Note

All claims expressed in this article are solely those of the authors and do not necessarily represent those of their affiliated organizations, or those of the publisher, the editors and the reviewers. Any product that may be evaluated in this article, or claim that may be made by its manufacturer, is not guaranteed or endorsed by the publisher.

## Abbreviations

HUS: hemolytic uremic syndrome
STEC: Shiga toxin-producing *Escherichia coli*
cHUS: complement mediated hemolytic uremic syndrome
PHIS: pediatric health information system
CHCA: child health corporation
ICD-9, ICD-10: International classification of diseases, ninth revision, tenth revision
CPT: correct procedures terminology
SD: standard deviation
OR: odds ratio
CI: confidence interval
FDA: food and drug administration
ICU: intensive care unit
LOS: length of stay
GI: gastro intestinal
TTP: thrombotic thromboctytopenic purpura.
