# Supplementary material for: Utilization Pattern for Eculizumab Among Children With Hemolytic Uremic Syndrome
Source: Front Pediatr. 2021 Oct 5;9:733042. doi: 10.3389/fped.2021.733042 (PMC8523981; doi:10.3389/fped.2021.733042)
Supplement: Supplementary file 1 [file Data_Sheet_1.PDF]

eTable 1 Diagnoses and Procedure codes

283.11 or D59.3 HUS

Generic drug code 164171- eculizumab

Any gastrointestinal condition

53100 - AGU w hemorrhage s obstr"
"53140 - CGU w hemor w/o obstruct"
"53190 - Gastric ulcer NOS"
"53240 - Chr DU w hemor w/o obstr"
"53250 - Chr DU w perf w/o obstr"
"53290 - DU NOS w/o comp"
"53340 - Chr peptic ulcer w hemor"
"53390 - Peptic ulcer NOS s comp"
"53510 - Atroph gastritis s hemor"
"53540 - Gastritis NEC w/o hemor"
"53550 - Gastroduodenitis NOS"
"53551 - Gastritis NOS w hemor"
"53560 - Duodenitis w/o hemor"
"53561 - Duodenitis w hemorrhage"
"5362 - Persistent vomiting"
"5363 - Gastroparesis"
"K561 - Intussusception"
"K562 - Volvulus"
"5550 - Reg enteritis sm intest"
"5552 - Reg enterit sm/lg intest"
"5559 - Regional enteritis NOS"
"5566 - Universal UC"
"5568 - Other ulcerative colitis"
"5569 - Ulcerative colitis NOS"
"5570 - Ac vasc insuff intestine"
"5579 - Vasc insuff intest NOS"
"55842 - Eosinophilic colitis"
"5589 - NonINF gastroent NEC&NOS"
"5600 - Intussusception"
"56962 - Colo/enterstmy mech comp"
"56981 - Intestinal fistula"
"56982 - Intestinal ulceration"

Specific Cardiac condition
Hypertesnion:
4019 - Hypertension NOS
40291 - HTN heart dis NOS w HF
40501 - Mal renovascular HTN
40509 - Mal secondary HTN NEC
40519 - Benign secondary HTN NEC
40591 - Renovascular HTN NEC
I10 - Essential hypertension
I110 - Hypertensive heart disease w heart failure
I119 - Hypertensive heart disease w/o heart failure
I150 - Renovascular hypertension
I151 - Hypertension secondary to renal disorders NEC
I152 - Hypertension secondary to endocrine disorders
I158 - Secondary hypertension NEC
I159 - Secondary hypertension NOS
I160 - Hypertensive urgency
I161 - Hypertensive emergency
CARDIOMYOPATHY: HYPERTROPHIC/ OBSTRUCTIVE
42511 - Hypertr obstr cardiomyop
42518 - Hypertr cardiomyop NEC
I421 - Obstructive hypertrophic cardiomyopathy
CARDIOMYOPATHY: DILATED
4254 - Prim cardiomyopathy NEC
4258 - Cardiomyopathy in DCE
4259 - 2nd cardiomyopathy NOS
4253 - Endocard fibroelastosis
I424 - Endocardial fibroelastosis
I427 - Cardiomyopathy D/T drug & external agent
I428 - Cardiomyopathies NEC
I429 - Cardiomyopathy NOS
I43 - Cardiomyopathy in DCE
I420 - Dilated cardiomyopathy
MYOCARDITIS
I400 - Infective myocarditis
42290 - Acute myocarditis NOS
42291 - Idiopathic myocarditis
42292 - Septic myocarditis
4290 - Myocarditis NOS
4280 - CHF NOS
4281 - Left heart failure
42820 - Systolic HF NOS
42821 - Acute systolic HF
42823 - Ac & chr systolic HF
42830 - Diastolic HF NOS
42840 - SYS & diastolic HF NOS
42841 - Ac SYS & diastolic HF
42843 - AcChr SYS & diastolic HF
4289 - Heart failure NOS
I501 - Left ventricular failure NOS
I5020 - Systolic heart failure NOS

I5021 - Acute systolic heart failure
I5031 - Acute diastolic heart failure
I5033 - Acute on chronic diastolic heart failure
I5040 - Systolic & diastolic heart failure NOS
I5041 - Acute systolic & diastolic heart failure
I5042 - Chronic systolic & diastolic heart failure
I50810 - Right heart failure NOS
I5082 - Biventricular heart failure
I509 - Heart failure NOS

PERICARDIAL DISEASE
4200 - Ac pericarditis in DCE
42090 - Acute pericarditis NOS
42091 - Ac idiopathic pericard
42099 - Acute pericarditis NEC
I300 - Acute nonspecific idiopathic pericarditis
I301 - Infective pericarditis
I309 - Acute pericarditis NOS
I32 - Pericarditis in DCE
I313 - Pericardial effusion
4230 - Hemopericardium
4233 - Cardiac tamponade
I314 - Cardiac tamponade
4238 - Pericardial disease NEC
4239 - Pericardial disease NOS
4232 - Constrictive pericard
I310 - Chronic adhesive pericarditis
I311 - Chronic constrictive pericarditis

ENDOCARDITIS
4210 - Ac/subac bact endocard
4219 - Ac/subac endocard NOS
42490 - Endocarditis unspecified
I330 - Acute & subacute infective endocarditis

HEART BLOCK
I440 - Atrioventricular block 1st degree
I441 - Atrioventricular block 2nd degree
I442 - Atrioventricular block complete
I4510 - Right bundle-branch block NOS
I4519 - Right bundle-branch block NEC
4260 - Complete A/V block
42610 - A/V block NOS
42613 - 2nd degree A/V block NEC
42652 - RBBB & LAFB

VENTRICULAR TACHYCARDIA/ ARRHYTHMIA
42741 - Ventricular fibrillation
4271 - PVT
I472 - Ventricular tachycardia
I4901 - Ventricular fibrillation
I4902 - Ventricular flutter

ATRIAL FLUTTER/ FIBRILLATION
42731 - Atrial fibrillation
42732 - Atrial flutter
I4892 - Atrial flutter NOS

SVT
4270 - PSVT
I471 - Supraventricular tachycardia

MITRAL VALVE DISEASE
I340 - Nonrheumatic mitral insufficiency
4240 - Mitral valve disorder

AORTIV VALVE DISEASE
I350 - Nonrheumatic aortic stenosis
I351 - Nonrheumatic aortic insufficiency
I352 - Nonrheumatic aortic stenosis w insufficiency
4241 - Aortic valve disorder

TRICUSPID VALVE DISEASE
I361 - Nonrheumatic tricuspid insufficiency
4242 - Nonrheum tricusp disord
PULMONARY VALVE DISEASE
4243 - Pulmonary valve disorder

Neurological condition
Seizures

SVT
4270 - PSVT
I471 - Supraventricular tachycardia

MITRAL VALVE DISEASE
I340 - Nonrheumatic mitral insufficiency
4240 - Mitral valve disorder

AORTIV VALVE DISEASE
I350 - Nonrheumatic aortic stenosis
I351 - Nonrheumatic aortic insufficiency
I352 - Nonrheumatic aortic stenosis w insufficiency
4241 - Aortic valve disorder

TRICUSPID VALVE DISEASE
I361 - Nonrheumatic tricuspid insufficiency

4242 - Nonrheum tricusp disord

PULMONARY VALVE DISEASE

4243 - Pulmonary valve disorder

Neurological condition

Seizures

34510 - Grand mal w/o intract
34511 - Grand mal w intract epil
3452 - Petit mal status
3453 - Grand mal status
34540 - LRE w CPS w/o intract
34550 - LRE w SPS w/o intract
34560 - Infant spasm w/o intract
34570 - Epil part cont s intract
34580 - Epilepsy NEC w/o intract
34590 - Epilepsy NOS w/o intract
34591 - Intractable epilepsy NOS
G40101 - LRSE & epileptic syndrome w SPS not intract w SE
G40109 - LRSE & epileptic synd w SPS not intract w/o SE
G40119 - LRSE & epileptic synd w SPS intract w/o SE
G40209 - LRSE & epileptic synd w CPS not intract w/o SE
G40219 - LRSE & epileptic syndrome w CPS intract w/o SE
G40301 - GIE & epileptic syndromes not intractable w SE
G40309 - GIE & epileptic syndromes not intractable w/o SE
G40401 - Oth gen epilepsy & epil synd not intract w SE
G40409 - Oth gen epilepsy & epil synd not intract w/o SE
G40801 - Epilepsy NEC not intract w status epilepticus
G40812 - Lennox-Gastaut syndrome not intract w/o SE
G40822 - Epileptic spasms not intractable w/o SE
G4089 - Seizures NEC
G40901 - Epilepsy NOS not intract w status epilepticus
G40909 - Epilepsy NOS not intract w/o status epilepticus
G40911 - Epilepsy NOS intractable w status epilepticus
G40A09 - Absence epileptic syndrome not intract w/o SE

Intracerebral hemorrhage :

4378 - Cerebrovasc disease NEC
430 - Subarachnoid hemorrhage
431 - Intracerebral hemorrhage
4320 - Nontraumatic EXDH
4321 - Subdural hemorrhage
4329 - Intracranial hemor NOS
I609 - Nontraumatic subarachnoid hemorrhage NOS
I615 - Nontraum intracerebral hemor intraventricular
I618 - Nontraumatic intracerebral hemorrhage NEC

I619 - Nontraumatic intracerebral hemorrhage NOS
I6200 - Nontraumatic subdural hemorrhage NOS
I6203 - Nontraumatic chronic subdural hemorrhage
I629 - Nontraumatic intracranial hemorrhage NOS
I63532 - CI D/T occl/sten NOS left post cerebral artery
I636 - Cereb infarct D/T cereb venous thromb nonpyogen

Brain infarct:
43301 - Basil art occl w infarct
43310 - Carotid occl s infarct
43311 - Carotid occl w infarct
43400 - Cereb thromb w/o infarct
43401 - Cereb thromb w infarct
43411 - Cerebral embol w infarct
43491 - Cereb art occl w infarct
4358 - Trans cereb ischemia NEC
4359 - Trans cereb ischemia NOS
436 - Acute ill-defined CVD
4371 - Ac cerebrovasc insuf NOS
I638 - Cerebral infarction NEC
I639 - Cerebral infarction NOS
I668 - Occlusion & stenosis cerebral arteries NEC
I669 - Occlusion & stenosis cerebral artery NOS
I6782 - Cerebral ischemia
I69354 - HEMI & hemipar follow CI affect lt non-dom side
I69392 - Facial weakness following cerebral infarction
I69398 - Sequelae cerebral infarction NEC
43301 - Basil art occl w infarct
43310 - Carotid occl s infarct
43311 - Carotid occl w infarct
43400 - Cereb thromb w/o infarct
43401 - Cereb thromb w infarct
43411 - Cerebral embol w infarct
43491 - Cereb art occl w infarct
4358 - Trans cereb ischemia NEC
4359 - Trans cereb ischemia NOS
436 - Acute ill-defined CVD
4371 - Ac cerebrovasc insuf NOS
I638 - Cerebral infarction NEC
I639 - Cerebral infarction NOS
I668 - Occlusion & stenosis cerebral arteries NEC
I669 - Occlusion & stenosis cerebral artery NOS
I6782 - Cerebral ischemia
I69354 - HEMI & hemipar follow CI affect lt non-dom side
I69392 - Facial weakness following cerebral infarction
I69398 - Sequelae cerebral infarction NEC
Encephalopathy
4372 - HTN encephalopathy
I674 - Hypertensive encephalopathy
34830 - Encephalopathy NOS

34831 - Metabolic encephalopathy
34839 - Encephalopathy NEC
34982 - Toxic encephalopathy
G92 - Toxic encephalopathy
G9340 - Encephalopathy NOS
G9341 - Metabolic encephalopathy
G9349 - Encephalopathy NEC
P9160 - Hypoxic ischemic encephalopathy NOS
P9163 - Severe hypoxic ischemic encephalopathy
P91819 - Neonatal encephalopathy NOS
I6783 - Posterior reversible encephalopathy syndrome

Anoxic Brain Injury
3481 - Anoxic brain damage
G931 - Anoxic brain damage NEC

Cerebral edema
3485 - Cerebral edema
G936 - Cerebral edema

Brain compression
3484 - Brain compression
G935 - Compression bra

Shiga toxin mediated (e.coli and shigella)

04141 - STEC O157
04142 - STEC NEC
04143 - STEC NOS
04149 - E. coli infect NEC & NOS
B9621 - Shiga toxin-producing E coli O157 as cause DCE
B9622 - Other Shiga toxin-producing E coli as cause DCE
B9623 - Shiga toxin-producing E coli NOS as cause DCE

Pneumococcal

0412 - Pneumococcus infect NOS
B953 - Streptococcus pneumoniae as cause DCE
481 - Pneumococcal pneumonia
48230 - Strep pneumonia NOS
48239 - Strep pneumonia NEC
0382 - Pneumococcal septicemia
A403 - Sepsis D/T Streptococcus pneumonia
3201 - Pneumococcal meningitis

Enteritis bacterial and viral:

0030 - Salmonella enteritis
0031 - Salmonella septicemia
0039 - Salmonella infection NOS
0040 - Shigella dysenteriae
0041 - Shigella flexneri
0043 - Shigella sonnei
0048 - Shigella infection NEC
0049 - Shigellosis NOS
00800 - E. coli NOS enteritis
00801 - Entpath E.coli enteritis
00802 - Enttox E. coli enteritis
00803 - Entinv E. coli enteritis
00804 - Enthemo E.coli enteritis
00809 - E. coli enteritis NEC
00841 - Staph enteritis
00842 - Pseudomonas enteritis

00843 - Campylobacter enteritis
00844 - Y. enterocol enteritis
00845 - C. difficile enteritis
00847 - Gram-neg enteritis NEC
00849 - Bacterial enteritis NEC
0085 - Bacterial enteritis NOS
0090 - Infectious enteritis NOS
0091 - Enteritis presum INF
0092 - Infectious diarrhea NOS
04141 - STEC O157
04142 - STEC NEC
04143 - STEC NOS
A030 - Shigellosis D/T Shigella dysenteriae
A032 - Shigellosis D/T Shigella boydii
A033 - Shigellosis D/T Shigella sonnei
A038 - Shigellosis NEC
A039 - Shigellosis NOS
A040 - Enteropathogenic Escherichia coli infection
A041 - Enterotoxigenic Escherichia coli infection
A042 - Enteroinvasive Escherichia coli infection
A043 - Enterohemorrhagic Escherichia coli infection
A044 - Intestinal Escherichia coli infections NEC
A045 - Campylobacter enteritis
A047 - Enterocolitis D/T Clostridium difficile
A0471 - Enterocolitis D/T Clostridium difficile recur
A0472 - Enterocol D/T Clostridium difficile not recur
A048 - Bacterial intestinal infections NEC
A049 - Bacterial intestinal infection NOS
A059 - Bacterial foodborne intoxication NOS
A09 - Infectious gastroenteritis & colitis NOS
A329 - Listeriosis NOS
B9620 - Escherichia coli NOS as cause DCE
B9621 - Shiga toxin-producing E coli O157 as cause DCE
B9622 - Other Shiga toxin-producing E coli as cause DCE
B9623 - Shiga toxin-producing E coli NOS as cause DCE
00861 - Rotavirus enteritis
00862 - Adenovirus enteritis
00863 - Norwalk virus enteritis
00869 - Viral enteritis NEC
0088 - Viral enteritis NOS
A080 - Rotaviral enteritis
A0819 - Acute gastroenteropathy D/T sm round viruses NEC
A082 - Adenoviral enteritis
A0832 - Astrovirus enteritis
A0839 - Viral enteritis NEC

A084 - Viral intestinal infection NOS

Sepsis/Shock
78552 - Septic shock
7907 - Bacteremia
99590 - SIRS NOS
99591 - Sepsis
99592 - Severe sepsis
99593 - SIRS D/T non-INF s organ
99594 - SIRS D/T non-INF w organ
B377 - Candidal sepsis
0031 - Salmonella septicemia
0362 - Meningococcemia
0369 - Meningococcal infect NOS
0380 - Streptococcal septicemia
03810 - Staph septicemia NOS
03811 - MSSA septicemia
03812 - MRSA septicemia
03819 - Staph septicemia NEC
0382 - Pneumococcal septicemia
0383 - Anaerobic septicemia
03840 - Gram-neg septicemia NOS
03841 - H. influenzae septicemia
03842 - E. coli septicemia
03843 - Pseudomonas septicemia
03849 - Gram-neg septicemia NEC
0388 - Septicemia NEC
0389 - Septicemia NOS
A021 - Salmonella sepsis
A400 - Sepsis D/T group A streptococcus
A401 - Sepsis D/T group B streptococcus
A403 - Sepsis D/T Streptococcus pneumoniae
A408 - Streptococcal sepsis NEC
A409 - Streptococcal sepsis NOS
A4101 - Sepsis D/T methicillin suscept staph aureus
A4102 - Sepsis D/T methicillin resistant staph aureus
A411 - Sepsis D/T specified staphylococcus NEC
A412 - Sepsis D/T staphylococcus NOS
A414 - Sepsis D/T anaerobes
A4151 - Sepsis D/T Escherichia coli
A4152 - Sepsis D/T Pseudomonas
A4153 - Sepsis D/T Serratia
A4159 - Gram-negative sepsis NEC
A4181 - Sepsis D/T Enterococcus
A4189 - Sepsis NEC
A419 - Sepsis organism NOS
A483 - Toxic shock syndrome
P3610 - Sepsis newborn D/T streptococci NOS
P3619 - Sepsis newborn D/T streptococci NEC
P362 - Sepsis newborn D/T Staphylococcus aureus
P3639 - Sepsis newborn D/T staphylococci NEC
P364 - Sepsis newborn D/T Escherichia coli
P368 - Bacterial sepsis newborn NEC
P369 - Bacterial sepsis newborn NOS
R6520 - Severe sepsis w/o septic shock
R6521 - Severe sepsis w septic shock

Shock

R570 - Cardiogenic shock<br>R571 - Hypovolemic shock<br>R578 - Other shock<br>R579 - Shock NOS

CKD<br>585 - Chronic renal failure<br>5851 - CKD-Stage I<br>5852 - CKD-Stage II<br>5853 - CKD-Stage III<br>5854 - CKD-Stage IV<br>5855 - CKD-Stage V<br>5859 - Chronic kidney dis NOS<br>5856 - ESRD<br>586 - Renal failure NOS<br>N181 - Chronic kidney disease stage 1<br>N182 - Chronic kidney disease stage 2<br>N183 - Chronic kidney disease stage 3<br>N184 - Chronic kidney disease stage 4<br>N185 - Chronic kidney disease stage 5<br>N186 - End stage renal disease<br>N189 - Chronic kidney disease NOS<br>N19 - Kidney failure NOS
**PROCEDURES**
**PLEURAL DRAINAGE**<br>32020 - INSERTION OF CHEST TUBE<br>32422 - THORACENTESIS W/TUBE INSERT<br>32551 - INSERTION OF CHEST TUBE<br>32556 - INSERT CATH PLEURA W/O IMAGE<br>32557 - INSERT CATH PLEURA W/ IMAGE<br>32554 - ASPIRATE PLEURA W/O IMAGING<br>32555 - ASPIRATE PLEURA W/ IMAGING<br>0B9N0ZZ - Drainage right pleura, open<br>0B9N30Z - Drainage right pleura w drainage device, perq<br>0B9P0ZZ - Drainage left pleura, open<br>0W29X0Z - Change drain device rt pleural cavity, external<br>0W9900Z - Drainage rt pleural cavity w drain device, open<br>0W990ZZ - Drainage right pleural cavity, open<br>0W9930Z - Drainage rt pleural cavity w drain device, perq<br>0W993ZZ - Drainage right pleural cavity, percutaneous<br>0W9940Z - Drainage rt pleur cavity w drain device, P-endo<br>0W9B0ZZ - Drainage left pleural cavity, open<br>0W9B30Z - Drainage lt pleural cavity w drain device, perq<br>0W9B3ZX - Drainage left pleural cavity, perq, diagnostic<br>0W9B3ZZ - Drainage left pleural cavity, percutaneous<br>0W9B40Z - Drainage lt pleur cavity w drain device, P-endo<br>0B9N0ZZ - Drainage right pleura, open<br>0B9N30Z - Drainage right pleura w drainage device, perq

Tracheostomy

51902 - Tracheostomy mech comp
V440 - Tracheostomy status
Z930 - Tracheostomy status

Gastrostomy

Z931 - Gastrostomy status
V441 - Gastrostomy status

Ileostomy

V442 - Ileostomy status
V443 - Colostomy status
V444 - Enterostomy status NEC
V4452 - Append-vesicostmy status
V453 - Intestinal bypass status
Z932 - Ileostomy status
Z934 - Artificial opening gastrointest tract status NEC

Dialysis and plasmapheresis

"3995 - Hemodialysis"
"5498 - Peritoneal dialysis"
"5A1D70Z - Performance urinary filtration, intermittent"
"5A1D90Z - Performance urinary filtration, continuous"
"5A1D80Z - Performance urinary filtration, prolong interm"

DIALYSIS CPT codes
"90935 - HEMODIALYSIS ONE EVALUATION"
"90935 - HEMODIALYSIS, ONE EVALUATION"
"90937 - HEMODIALYSIS REPEATED EVAL"
"90937 - HEMODIALYSIS, REPEATED EVAL"
"90945 - DIALYSIS ONE EVALUATION"
"90945 - DIALYSIS, ONE EVALUATION"
"90947 - DIALYSIS REPEATED EVAL"
"90947 - DIALYSIS, REPEATED EVAL"
"90997 - HEMOPERFUSION"
"90999 - DIALYSIS PROCEDURE"
"C1750 - Long-term dialysis cath"
"C1751 - Periph infusion cath"
"C1752 - Short-term dialysis cath"

excl_plasmaphar (all procedure codes)

"9971 - Ther plasmapheresis"

"9972 - Ther leukopheresis"

"9973 - Ther erythrocytapheresis"

"9974 - Ther plateletpheresis"

"9976 - Extracorp immunoadsorp"

"9977 - Appl adhes barrier subst"

"9979 - Ther apheresis NEC"
