# Supplementary material for: Utilization Pattern for Eculizumab Among Children With Hemolytic Uremic Syndrome
Source: Front Pediatr. 2021 Oct 5;9:733042. doi: 10.3389/fped.2021.733042 (PMC8523981; doi:10.3389/fped.2021.733042)
Supplement: Supplementary file 2 [file Data_Sheet_2.PDF]

eTable 2: **Clinical factors associated with eculizumab administration on index hospitalization**

	Individual GI, Cardiac, and neurologic Conditions Model 1			Grouped GI, Cardiac, and neurological Conditions Model 2			Additive Effect of Grouped GI, Cardiac, and neurological Conditions[a] Model 3		
Variable	OR	95% CI	p-value	OR	95% CI	p-value	OR	95% CI	p-value
Age									
<1 year	0.71	(0.38,1.32)	0.277	0.75	(0.41,1.38)	0.360	0.76	(0.42,1.38)	0.368
1 to <5 years	1.32	(0.71,2.46)	0.376	1.47	(0.80,2.67)	0.211	1.41	(0.78,2.55)	0.251
>=5 years	ref	ref	ref	ref	ref	ref	ref	ref	ref
Race									
White	ref	ref	ref	ref	ref	ref	ref	ref	ref
Black	0.91	(0.44,1.89)	0.799	0.96	(0.47,1.97)	0.909	0.98	(0.48,1.98)	0.945
Asian	1.99	(0.81,4.88)	0.132	2.08	(0.86,5.02)	0.103	2.09	(0.88,4.95)	0.095
Other	1.29	(0.80,2.10)	0.295	1.34	(0.84,2.14)	0.220	1.38	(0.87,2.19)	0.174
Male	0.80	(0.55,1.16)	0.240	0.77	(0.53,1.10)	0.154	0.77	(0.54,1.11)	0.161
Plasmapheresis	1.86	(0.91,3.79)	0.087	1.86	(0.94,3.66)	0.073	2.00	(1.03,3.90)	0.041
Dialysis	1.18	(0.79,1.77)	0.424	1.14	(0.76,1.70)	0.520	1.11	(0.75,1.65)	0.600
Sepsis	0.85	(0.47,1.54)	0.589	0.85	(0.48,1.51)	0.579	0.82	(0.47,1.44)	0.494
Shock	1.98	(0.90,4.33)	0.088	2.48	(1.20,5.13)	0.014	2.41	(1.17,4.96)	0.017
GI Surgery	1.58	(0.50,5.02)	0.440	1.68	(0.59,4.83)	0.332	1.86	(0.66,5.18)	0.239
Pleural Drain	1.91	(0.77,4.71)	0.163	2.06	(0.88,4.83)	0.095	1.92	(0.83,4.46)	0.128
Endotracheal Tube	1.21	(0.72,2.03)	0.469	1.29	(0.78,2.11)	0.318	1.50	(0.92,2.45)	0.101
Shiga	1.04	(0.52,2.06)	0.915	1.15	(0.59,2.23)	0.677	1.18	(0.61,2.27)	0.629
Pneumococcal	0.72	(0.27,1.95)	0.521	0.66	(0.25,1.70)	0.385	0.72	(0.28,1.85)	0.490
Not Enteritis	3.65	(1.93,6.88)	<0.001	3.66	(1.96,6.84)	<0.001	3.69	(1.98,6.87)	<0.001
Any GI condition[b]	1.48	(1.00,2.18)	0.050	1.45	(0.99,2.12)	0.058			
Hypertension	2.00	(1.36,2.94)	<0.001						
Myocarditis/Cardiomyopathy/	6.77	(3.16,14.54)	<0.001						

Congestive Heart Failure									
Other Cardiac Condition[b]	1.30	(0.68,2.50)	0.424						
Seizure	2.72	(1.47,5.04)	0.001						
Encephalopathy	3.44	(1.97,6.00)	<0.001						
Cerebral Edema/Brain Compression	1.23	(0.39,3.87)	0.723						
Other neurologic Condition	1.05	(0.51,2.18)	0.888						
Any Cardiac Condition				2.63	(1.79,3.86)	<0.001			
Any neurologic Condition[b]				4.14	(2.68,6.38)	<0.001			
Number of GI, cardiac, neurologic condition categories									
0							ref	ref	ref
1							3.00	(1.81,4.97)	<0.001
2							5.79	(3.35,10.01)	<0.001
3							15.69	(7.50,32.81)	<0.001

GI: gastro intestinal

[a] A variable was constructed to evaluate the potential additive effect of having multiple conditions among cardiac, neurologic, and gastrointestinal categories. This variable was constructed by adding 3 binary variables indicators for “any neurologic condition,” “any cardiac condition,” and “any gastrointestinal” condition.

[b] “Other Cardiac Condition” includes pericardial disease, endocarditis, cardiac arrest, valve disorders, arrhythmia, and heart block. “Other neurological Condition” includes meningitis, intracerebral hemorrhage, cerebral infarct, hemiplegia, and anoxic brain. Any GI condition includes gastrointestinal ulceration, hemorrhage, volvulus, intussusception, inflammatory or ischemic bowel disease, eosoniphilic colitis, ileostomy or fistulas, gastro paresis, persistent vomiting, liver or gall bladder disease
